# WEDM Used for Machining High Entropy Alloys

**DOI:** 10.3390/ma13214823

**Published:** 2020-10-28

**Authors:** Katerina Mouralova, Libor Benes, Radim Zahradnicek, Josef Bednar, Antonin Zadera, Jiří Fries, Vaclav Kana

**Affiliations:** 1 Faculty of Mechanical Engineering, Brno University of Technology, 616 69 Brno, Czech Republic; zahradnicek@vutbr.cz (R.Z.); bednar@fme.vutbr.cz (J.B.); zadera@fme.vutbr.cz (A.Z.); kana@fme.vutbr.cz (V.K.); 2 Faculty of Mechanical Engineering, Czech Technical University in Prague, 166 07 Prague, Czech Republic; libor.benes@ujep.cz; 3 Department of Production Machines and Design, Technical University of Ostrava, 708 33 Ostrava, Czech Republic; jiri.fries@vsb.cz

**Keywords:** WEDM, wire electrical discharge machining, high entropy alloy, design of experiment, machining parameters

## Abstract

Unconventional wire electrical discharge machining technology (WEDM) is a key machining process, especially for machining newly emerging materials, as there are almost no restrictions (only at least minimal electrical conductivity) in terms of demands on the mechanical properties of the workpiece or the need to develop new tool geometry. This study is the first to present an analysis of the machinability of newly developed high entropy alloys (HEAs), namely FeCoCrMnNi and FeCoCrMnNiC_0.2_, using WEDM. The aim of this study was to find the optimal setting of machine parameters for the efficient production of parts with the required surface quality without defects. For this reason, an extensive design of experiments consisting of 66 rounds was performed, which took into account the influence of five input factors in the form of pulse off time, gap voltage, discharge current, pulse on time, and wire speed on cutting speed and the quality of the machined surface and its subsurface layer. The analysis of topography, morphology, subsurface layers, chemical composition analysis (EDX), and lamella analysis using a transmission electron microscope (TEM) were performed. An optimal setting of the machine parameters was found, which enables machining of FeCoCrMnNi and FeCoCrMnNiC_0.2_ with the required surface quality without defects.

## 1. Introduction

Unconventional wire electrical discharge machining technology (WEDM), which removes material through the thermoelectric process, is indispensable in many areas of industry. The removal of materials takes place between the workpiece and the tool electrode by means of periodically repeating electrical pulses with a duration of 10^−4^ up to 10^−6^ s [1]. These electrical impulses are generated by the machine generator and cause the removal of material not only by electrical discharge, but also by evaporation. The eroded material is always washed away from the cutting point by a stream of dielectric liquid, a liquid with high electrical resistance [2,3]. Because of the fact that, between the tool and the workpiece, in the form of a wire electrode (a wire with a diameter from 0.02 to 0.3 mm, most often made of brass), there is always the so-called kerf; the workpiece is not loaded by conventional forces, as is the case with conventional machining methods. For this reason, it is possible to machine even very soft materials or create thin-walled profiles. Another advantage is the fact that all at least minimally electrically conductive materials can be machined, regardless of their toughness or hardness, which can also prevent conventional machining of these materials. The disadvantage of the process is its higher energy consumption as well as the relatively slow removal of material [4,5].

High entropy alloys (HEAs) are formed by mixing the same or approximately a large proportion of five or more elements. HEAis currently a very promising material, and is the focus of many scientists, especially in the field of mechanical engineering. Current research shows that some HEAs have a significantly better strength-to-weight ratio or higher fracture toughness than conventional alloys [6].

WEDM, like any technological process, needs to be optimized to achieve the most efficient machining possible. In the case of WEDM, it is mainly about optimizing the settings of machine parameters to maximize the cutting speed (i.e., material removal rate, MRR) to reduce machine times, and thus reduce the energy intensity of the technological operation. However, this optimization must also take into account the fulfilment of other conditions, which are the maximization of surface quality in the form of topography as well as the occurrence of defects in the surface or subsurface layer. In order to optimize WEDM, many designs of experiments and studies have been performed to streamline machining of, for example, aluminium alloy 7475-T7351 [7], Ampcoloy copper alloy [8], Creusabro steel [9], Hardox [10], or Hadfield steel [11]. The aim of this study was to machine the very first newly invented high entropy alloys, while machining (conventional or unconventional) of any material from the HEA group has been never presented in any study. The results of this study will, therefore, provide the very first knowledge about the machinability of two high-entropy alloys. HEAs also have very promising possibilities of use for nuclear reactors, where they would clearly exceed the service life of currently used stainless steels [12].

### Literature Review

Guo [13] studied the machining of high-entropy alloys that have superior low-temperature mechanical properties, although, because of its low surface quality, it requires post-machining. For the experiments, they chose a selective laser melting CoCrFeMnNi high-entropy alloy and machined it employing commonly used thermal, mechanical, and electrochemical machining processes. Smoother surfaces were achieved as the result of the WEDM process, which were subsequently mechanically polished to obtain an extra smooth surface. Shen [14] studied high-entropy nitride coatings using the machining processes and discovered their high hardness and superior oxidation resistance, which provides them with big potential in hard coating applications. Specifically, they focused on the adhesion strength, thermal stability, and cutting performance in their experiments. He [15] focused on the possibility to precipitate the nanosized coherent reinforcing phase, during which they obtained extraordinary balanced tensile properties of the studied high-entropy alloy using room temperature. They were able to demonstrate the usage of integrated strengthening approaches for the manipulation of the fcc-HEA systems properties. Pickering [16] worked with the material of CrMnFeCoNi, which is considered to be an exemplar high-entropy alloy. It is stable at all temperatures below the melting point. However, it was proved during the experiments that it is not thermodynamically stable as a single phase at all of the temperatures. Zhang [17] studied the polymorphism in a high-entropy alloy, that is, if it is still possible because of the entropy-stabilized forms of crystalline. However, a polymorphic transition was revealed during the in situ high-pressure synchrotron X-ray diffraction from the face-centred-cubic to hexagonal-close-packing structure in the high-entropy alloy. Laplanche [18] studied the microstructure evolution and critical stress in the CrMnFeCoNi high-entropy alloy. They performed tensile tests using the room temperature and liquid nitrogen and the interruptions at various strains in order to assess the microstructure evolution by transmission electron microscope (TEM). In addition to that, twin widths with the spaces, dislocation densities, and volume fractions were defined during the experiments. Yao [19] focused on carbothermal shock synthesis of high-entropy alloy nanoparticles in their research. They developed a method for producing nanoparticles with up to eight various elements and they were successful in creating PtPdRhRuCe nanoparticles, i.e., high-entropy alloy nanoparticles for the catalyzation of ammonia oxidation. Otto [20] studied the decomposition of a single-phase high-entropy alloy subsequently to rather long anneals of 500 days using intermediate temperatures of 500–900 °C, which afterwards ledto the establishment of thermodynamic equilibrium. Tracy [21] chose a high-entropy alloy CrMnFeCoNias the experimental material for their research, which has superior properties compared with conventional alloys. They focused on the high-pressure synthesis of a hexagonal close-packed phase of the chosen alloy that revealed the structures and properties tuning unreachable with the help of traditional processing techniques. Wani [22] investigated the AlCoCrFeNi_2.1_high-entropy alloy and their mechanical properties and the microstructure development being heavily cold-rolled and annealed. The material annealed displayed a combination of remarkable strength-ductility and the ultrafine structure, which can also be thermos-mechanically processed.

## 2. Experimental Setup and Material

### 2.1. Experimental Material

**Figure 1:**
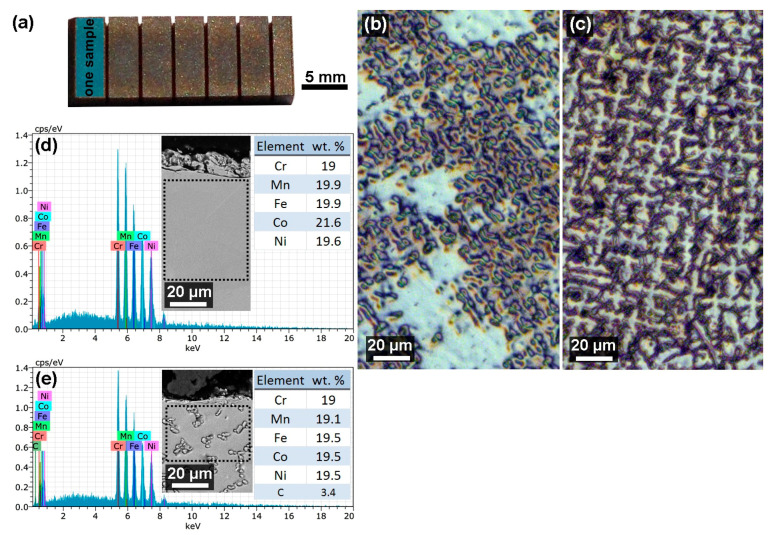
(**a**) An example of produced samples within the experiment with marking of the surface of one sample; (**b**) microstructure of 1Fe material; (**c**) microstructure of 1Fe0.2C material; (**d**) the analysis of the chemical composition of 1Fe material at a given place according to the scanning electron microscope (SEM) image; (**e**) the analysis of the chemical composition of 1Fe0.2C material at a given location according to the SEM image.

The samples for the experiment shown in Figure 1a were made of two types of high entropy alloys with the chemical composition according to Table 1. Thus, a total of 33 samples were made of 1Fe alloy and 33 samples of 1Fe0.2C alloy. These two alloys, FeCoCrMnNi and FeCoCrMnNiC_0.2_, were designed and cast into an ingot shape according to the Nagas study [23]; the microstructure of both materials is shown using light microscopy (LM) in Figure 1b. HEAs have a significantly better strength-to-weight ratio and higher fracture toughness and tensile strength than conventional alloys. They are also highly corrosion-resistant. Vickers hardness is for FeCoCrMnNi—146 HV alloy and for FeCoCrMnNiC0.2—249 HV alloy. A starting semi-product with a thickness of 10 mm was used for the experiment, with the length of the section of every sample being 3 mm each.

### 2.2. WEDM Machine Setup

A wire electrical discharge machine of the EU64 type supplied by the manufacturer MAKINO (Tokyo, Japan) was used for the production of samples of the design of experiments (DoE). This WEDM machine was equipped with computer numerical control (CNC) control of all axes. In order to efficiently cool and ensure a continuous supply of dielectric fluid to the cutting point, the workpiece was completely immersed in a dielectric bath represented by deionized water throughout the machining process. The brass wire marked CUT E supplied by PENTA TRADING (Prague, Czech Republic) was used for the machining. This wire was composed of 60%copper and 40%zinc, with a diameter of 0.25 mm.

The design of experiments is used to test complex tasks, for which the final result is given by a combination of many factors. The method is thus based on statistical testing of the influence of individual factors and their powers and interactions on the monitored output variables (responses). Thanks to the systematic selection of different values (levels) of factors, the information on responses can be obtained very efficiently, including mathematical models, where only statistically significant factors appear. The DoE method thus significantly reduces the number of tests required to understand and describe the process. It is mostly used in the field of research and development during testing and validation of new products or systems. In this study, a design of experiments was performed, which was based on monitoring the influence of five independent parameters of the machine settings. These were pulse off time (*T_off_*), gap voltage (*U*), discharge current (*I*), pulse on time (*T_on_*), and wire speed (*v*), while the limit values of individual parameters are written in Table 2 and have been determined on the basis of extensive previous tests [24].

Specifically, a “response surface design” was used, which describes the action of individual factors and all quadratic members of the input factors, i.e., the interactions and quadrates of these factors. This design is obtained by the composition:

- “Half factorial design 2^5-1^”, this design is created as a systematic selection of half of the vertices of a five-dimensional cube representing the investigated space of input variables;
- 10 axial points, which represent the centres of the walls of this five-dimensional cube in the input variables;
- 7 centre points, which represent repeated measurements in the centre of this five-dimensional cube of input variables, are used to capture the repeatability of the experiment and to describe the curvature of the response area.

This experimental plan is called “central composite design”, and the individual rounds are listed in Table 3, where it can also be seen that the individual rounds were randomized to eliminate systematic errors caused by the order of measurement. This data collection plan is described in detail, for example, in Montgomery [25].

## 3. Results and Discussion

### 3.1. Experimental Methods

In order to remove common impurities from the surface of the samples, they were placed in an ultrasonic cleaner for 20 min and cleaned before the observation. The study of the surface and subsurface layer was performed using a Lyra3 type electron microscope from TESCAN (Brno, Czech Republic), which was also equipped with, among other things, an energy-dispersive X-ray detector (EDX). In order to study the subsurface layer, metallographic preparations were made from all samples, which enabled the study of their crosssections. These preparations were prepared by conventional techniques—wet grinding and diamond paste polishing using the Tegramin30 automatic preparation system supplied by STRUERS (Westlake, Cleveland, OH, USA). The final mechanical-chemical finishing was performed with the OP-Chem suspension also from the company STRUERS. After etching with HCL/HNO_3_/H_2_O in a ratio of 3:1:2, while the 1Fe material was etched for 20 s and the 1Fe0.2C material was etched for 60 s, the samples were studied both by electron microscopy and using an Axio Observer Z1m light microscope from ZEISS (Jena, Germany). The evaluation of the topography of the machined surfaces (including images of 3D reflections) was performed with the help of a contact 3D profilometer Dektak XT supplied by the manufacturer BRUKER (Billerica, MA, USA). The measured data were further processed in Vision 64 and Gwyddion (5.1) software. Using a Helios-type electron microscope from THERMO FISHER (Hillsboro, OR, USA), which was also equipped with a focused ion beam (FIB), a lamella was prepared to study the material composition using EDX in a transmission electron microscope of the Titan type from THERMO FISHER.

### 3.2. Statistical Evaluation of the Cutting Speed

**Figure 2:**
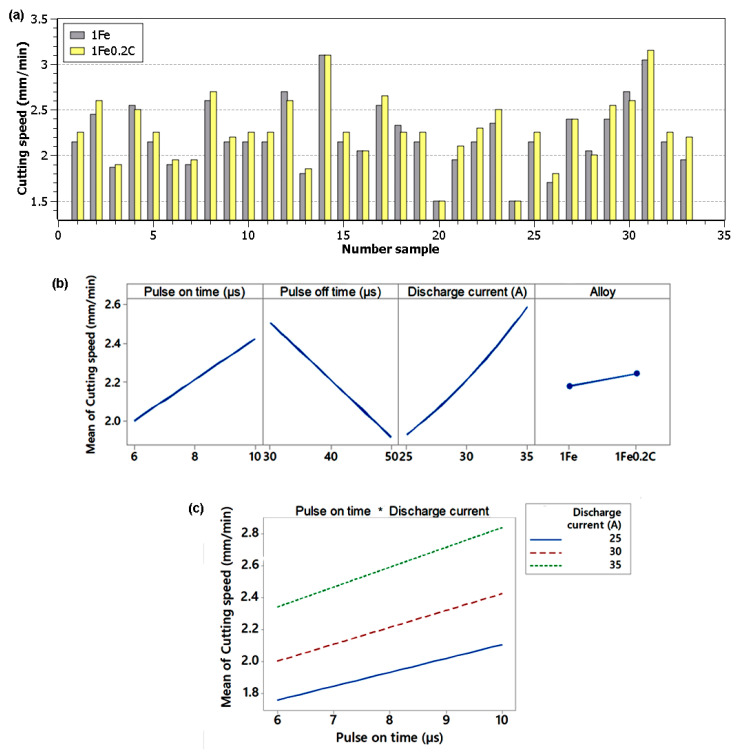
(**a**)The cutting speed of individual samples of 1Fe and 1Fe0.2C material, (**b**) the main effect plot for cutting speed, (**c**) the interaction plot for cutting speed.

Unconventional electrical discharge machining technology works quite differently from conventional technologies in terms of the cutting speed. Here, it is not possible to enter the required cutting speed when programming the machine. However, it is completely created and influenced by a set of machine parameter settings, while during machining, it is always shown on the display of the given device. This speed is generated by the machine so that the tool (wire electrode) and the workpiece never come into contact, so that there is always a so-called kerf between them. The cutting speed, which was read from the machine display during machining of the individual experimental samples, was plotted into the graph and is shown in Figure 2a. The machining speed ranged from a speed of 1.5 mm/min to a speed of 3.15 mm/min, which was achieved when machining Sample 31 from the material 1Fe0.2C with the setting of parameters *U* = 50 V, *T_on_* = 10 µs, *T_off_* = 30 µs, *v* = 14 m/min, and *I* = 35 A. Because of the fact that no high entropy alloy has been machined by any conventional or unconventional technology so far, it is not possible to compare these cutting speeds with other studies. However, it can be said that this speed was higher than when machining Hastelloy X [26] and, conversely, lower than when machining Hadfield steel [11] with the same thickness of 10 mm. Furthermore, it can also be stated that, in most cases, with the setting of the same machine parameters, a higher cutting speed was recorded when machining 1Fe0.2C alloy than when machining 1Fe; this difference is statistically significant. The wire electrode did not break during machining of any of the samples.

Based on the measured outputs, a full quadratic regression model was constructed, which describes the cutting speed. Insignificant predictors (*p*-value ≥ 0.05), including insignificant interactions and quadrates, were removed from the model using the “stepwise selection” method. The coefficient of determination R^2^ = 97.7%, i.e., the model describes 97.7% of the variability of the monitored cutting speeds. The model is adequate (*p*-value_Lack-of-Fit_ = 0.225), which means that no significant factors were involved during the experiment that were not part of the experiments. Table 4 shows the contributions of individual predictors, including the *p*-value test of the significance of individual predictors. The predictor is significant with *p*-value < 0.05.

Graphically, the effect of individual predictors on the cutting speed is shown in Figure 2b,c. From the main effect plot, it is clear that pulse on time and discharge current have a positive effect on the cutting speed and a negative effect on pulse off time; the type of alloy itself (1Fe or 1Fe0.2C) is not essential in comparison with these factors, although its effect is statistically significant. From the only significant interaction, it is clear that the cutting speed increases faster depending on the pulse on time at a higher current than at a lower current.

The regression Equation (1) itself describes the dependence of the cutting speed on the input parameters and the type of alloy 1Fe or 1Fe0.2C. For individual alloys, they differ only by the additive constant, which means that the shape of the response surfaces is the same for both alloys and these surfaces are only shifted.

(1) $$\begin{array}{l} v_{c}=const_{alloy}-0.0069T_{on}-0.02944T_{off}-0.0785I+0.001904I^{2}+0.00375T_{on}I,\\ where\;\;const_{alloy}=\left\{ \begin{array}{l} 3.157forAlloy1Fe,\\ 3.219forAlloy1Fe0.2C. \end{array}\right. \end{array}$$

### 3.3. Statistical Evaluation of Sample Surface Topography

The concept of quality in terms of the production technology must be understood as the accuracy of dimensions, accuracy of geometric shape, surface, and surface roughness. The surface roughness, i.e., the topography or surface microgeometry and the properties of the surface layer, significantly affect the service life and reliability of the operation of the components. The running accuracy of machine parts, their noise, running-in time, friction losses, wear resistance, and so on depend on the topography. Therefore, in this large experiment, the parameters of two topography parameters were evaluated: the arithmetical mean deviation of profile (Ra) and the maximum height of profile (Rz). These, including 3D reliefs, were evaluated using a Dektak XT contact 3D profilometer according to the corresponding standard for profile parameters ISO 4287 [27]. Five randomly selected places on each sample were measured and then averaged from these values.

**Figure 3:**
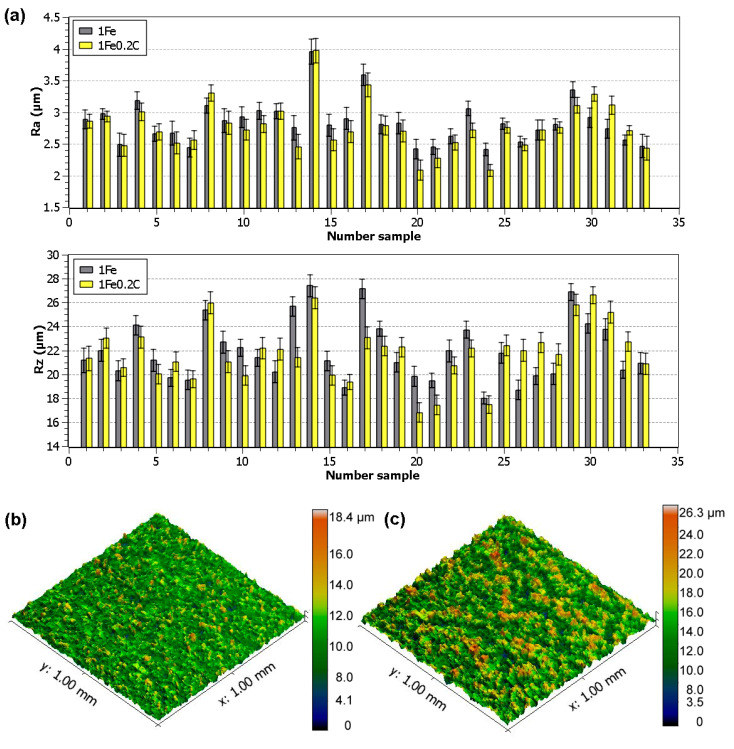
(**a**) The evaluated surface topography parameters Ra and Rz of individual experimental samples, (**b**) 3D relief of Sample 20 made of 1Fe0.2C, (**c**) 3D relief of Sample 14 made of 1Fe alloy.

The measured values of the topography parameters Ra and Rz of all 66 machined samples were compiled into the graphs shown in Figure 3a, with a standard deviation for each measurement. The lowest parameters Ra and Rz were simultaneously achieved in the sample made of 1Fe0.2C alloy according to the settings for Sample 20 (*U* = 70 V, *T_on_* = 6 µs, *T_off_* = 50 µs, *v* = 14 m/min, and *I* = 25 A), i.e., Ra 2.08 µm. A 3D relief of this surface is shown in Figure 3b, wherein, when compared with the relief of the sample with the highest topography parameters in Figure 3c, there is a clear difference, especially in the depth and frequency of occurrence of individual craters. Similar values of the surface topography were evaluated for samples with a thickness of 10 mm also from the material SAE 4140 steel [28], Ti-6Al-4V, or Inconel 718 [29].

**Figure 4:**
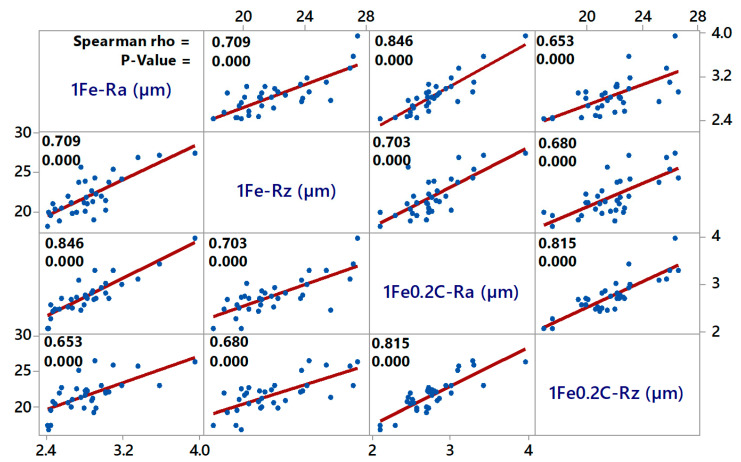
Matrix plot of surface topography parameters Ra and Rz.

The above-mentioned topography parameters for both alloys are interrelated, as can be seen from the graph in Figure 3a, and from the following matrix plot (Figure 4), where, for each scatter plot, the Spearman rank correlation coefficient and the *p*-value for the independence test are given. The axis descriptions of the individual sub-graphs are located on the diagonal of the graph, as used in the matrix plot. Spearman’s coefficient of the order correlation is statistically better than the commonly used Pearson’s correlation coefficient because, in the test of independence, it is not necessary to assume that the data come from a two-dimensional normal distribution. As *p*-value = 0.000 < 0.05, we always reject the hypothesis of independence and all characteristics can be considered positively dependent.

As in the case of the cutting speed, regression models were constructed for Ra and Rzfor both alloys. This time, the response surfaces for the individual alloys were obtained differently (they were not only shifted as for the cutting speed), and even different interactions were obtained. The contributions of individual interactions to the overall variability of surface topographic parameters are in percentage units. Because there are 14 second-order interactions in the model and they are tested at a significance level of 0.05, with a first-species error of 5% allowed, there is a relatively high probability that we will declare a significant interaction that is not really significant. Therefore, for clarity, Table 5 shows only the contributions of individual input variables and second-order interactions are presented as a whole. The first line shows the percentages of the variability of a given surface topography characteristic described by the model, i.e., the coefficients of determination. The second line shows the contributions of the variability of all linear terms and the last line is the contribution of the variability of second-order interactions as a whole. From the above table, it is clear that the input parameters pulse on time and discharge current have the greatest influence.

**Figure 5:**
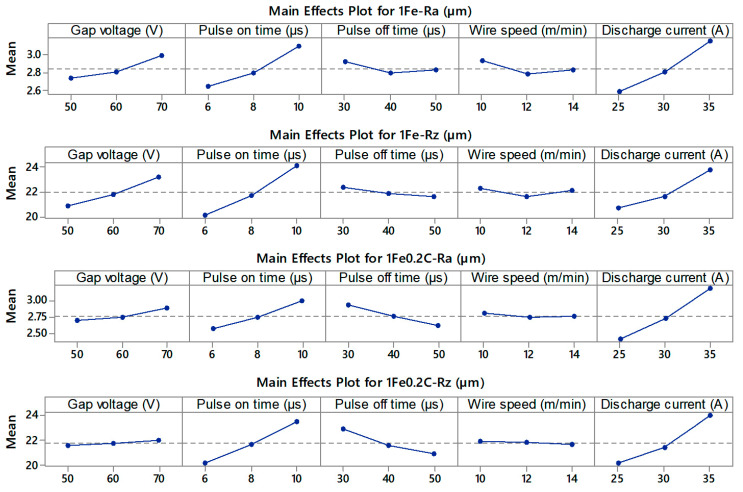
Main effect plot of Ra and Rz for both alloys.

For the sake of greater clarity, the individual equations are not given, but the effect of individual factors on the characteristics of the surface topography is graphically nationalized using the main effect plot shown in Figure 5.

### 3.4. Morphology of Machined Surfaces, Analysis of Chemical Composition, and Subsurface Area

The morphology of the electrical discharged machined surface is specific and very irregular compared with conventionally machined surfaces. Because the surface morphology is not created by the contact with the tool, the resulting surface is formed by a large number of randomly oriented craters formed during erosion. During the eroding process, several micrometres of the workpiece surface are completely melted because of the action of temperatures of 10,000–20,000 °C [30]. However, in the order of microseconds, this molten surface is cooled by a stream of dielectric fluid. The resulting so-called recast layer, which is characteristic for the electric discharge machining, has been studied in virtually all machined materials (e.g., Inconel 718 [31], Titanium [32], AISI H13 tool steel [33], or HSLA steel [34]) to a greater or lesser extent measure. In addition, this layer contains diffused elements from the wire electrode, because the effects of very high temperatures were also intense diffusion processes between the tool electrode and the workpiece. Diffusion phenomena have been described in many studies, such as Huang [35], Klocke [36], or Kumar [37]. The resulting surface morphology is a key aspect in predicting the correct functionality and life of manufactured components. If defects in the form of cracks (Mouralova [7]) or burnt cavities (Mouralova [11]) occur in the surface or subsurface area, this will mean a possible malfunction of the part or a reduction in its service life. The study of the surface morphology of all machined samples was performed using a Lyra3 electron microscope. In all cases, a backscattered electron (BSE) detector was used for imaging, and the samples were always studied at a magnification of 1000×, 2500×, and then 4000×.

**Figure 6:**
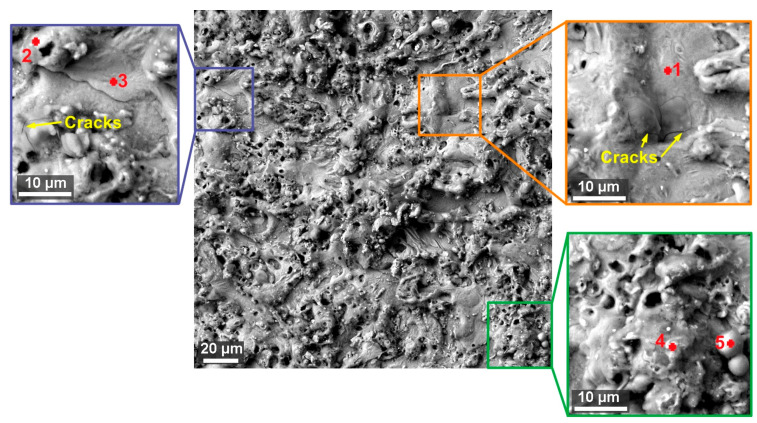
Surface morphology of Sample 20 (machined by setting machine parameters: *U* = 70 V, *T_on_* = 6 µs, *T_off_* = 50 µs, *v* = 14 m/min, and *I* = 25) made of 1Fe alloy (SEM/backscattered electron (BSE)) including details and marking of places where energy-dispersive X-ray detector (EDX) analysis was performed, the results of which are in Table 6.

**Figure 7:**
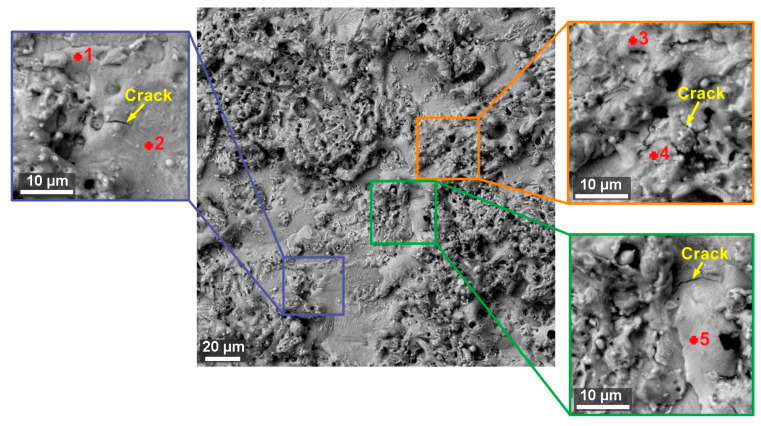
Surface morphology of Sample 20 (machined by setting machine parameters: *U* = 70 V, *T_on_* = 6 µs, *T_off_* = 50 µs, *v* = 14 m/min, and *I* = 25 A) made of 1Fe0.2C alloy (SEM/BSE) including details and marking of places where EDX analysis was performed, the results of which are in Table 7.

The morphology of all samples was carefully examined, and it was discovered that small cracks up to 10 µm in length appeared on the surface of all samples from both alloys. However, these cracks are only occasional, and their nature is shown in Figure 6 for 1Fe alloy and in Figure 7 for 1Fe0.2C alloy. A similar surface on which such small cracks were discovered was studied when machining Ampcoloy copper alloy [8]. In the samples of 1Fe alloy, approximately 30% of the surface is formed by smooth bottoms of craters, where there was no significant diffusion of elements from the wire electrode and their content is a maximum of 3.3 wt.% of copper and 2.1 wt.% of zinc (see Table 6 and Table 7). However, some places were not contaminated with zinc of the wire electrode at all, as can be seen from the measurement Place 1 in Figure 6. The samples made of 1Fe0.2C alloy were covered with a significantly larger number of smooth places, which were created for about 60% of the entire surface. Furthermore, on the surface of the place, there is a large amount of recast material, which is composed of a significantly larger amount of diffused elements from the wire electrode, where this content is already up to 20.3 wt.% of copper and 5.7 wt.% of zinc. The difference between the amount of diffused copper and zinc from the wire electrode (in the wire electrode, the proportion of these elements is 60% of Cu and 40% of Zn) is due to the different solubility of copper and zinc in HAEs, and because, unlike zinc, copper forms a stable intermetallic phase (copper is one of the alloying elements). A similar reduced zinc diffusion ratio has been studied on other WEDM machined materials such as Hadfield steel [11].

**Figure 8:**
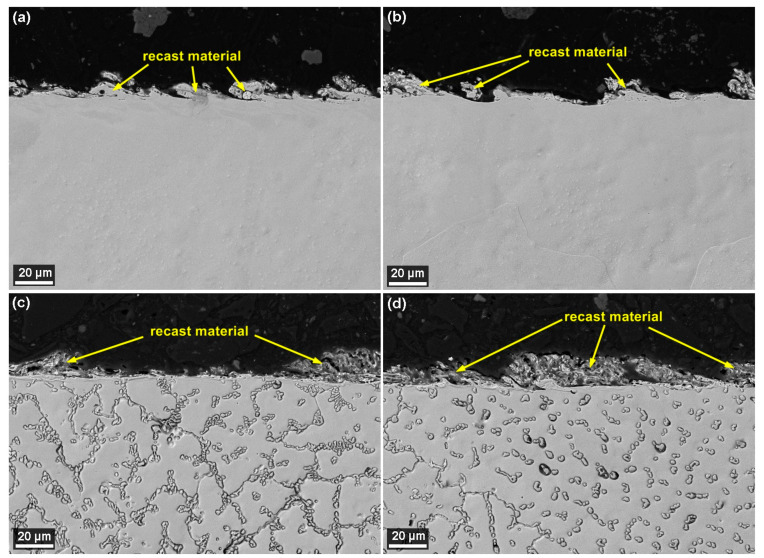
Crosssections of the SEM/BSE sample: (**a**) Sample 20 made of 1Fe alloy machined by the setting of the machine parameters: *U* = 70 V, *T_on_* = 6 µs, *T_off_* = 50 µs, *v* = 14 m/min, and *I* = 25 A; (**b**) Sample 14 made of 1Fe alloy machined by the setting of machine parameters: *U* = 70 V, *T_on_* = 10 µs, *T_off_* = 30 µs, *v* = 10 m/min, and *I* = 35 A; (**c**) Sample 20 made of 1Fe0.2C alloy machined by the setting of machine parameters: *U* = 70 V, *T_on_* = 6 µs, *T_off_* = 50 µs, *v* = 14 m/min, and *I* = 25 A; (**d**) Sample 14 made of 1Fe0.2C alloy machined by the setting of the machine parameters: *U* = 70 V, *T_on_* = 10 µs, *T_off_* = 30 µs, *v* = 10 m/min, and *I* = 35 A.

The examination of the condition of the subsurface area of the samples was made possible thanks to the production of metallographic preparations and their subsequent examination using electron microscopy. Crosssections were observed on a Lyra3 device, using a backscattered electron detector, each with a magnification of 1000× and then 2500× and 4000×. The analysis of the subsurface state of the machined layer is always crucial, especially in terms of the occurrence of defects, which are usually not visible when viewed from above. However, if we look at the cross-sectional images of the samples of both alloys shown in Figure 8, it is clear from them that there are no cracks or burnt cavities. This defect-free state was also studied on all other samples made of both alloys. It is thus clear that the small cracks visible from above (Figure 6 and Figure 7) on the machined surfaces do not interfere in any way with the depth of the material, and thus do not affect the service life and the correct functionality of the manufactured parts. From Figure 8, it is further apparent that each alloy has a different character and shape of recast material. The thickness of the recast layer was smaller in the samples made of 1Fe and did not exceed 20 µm. The layer was not continuous; it was only an occurrence on about 50% of the surface. For samples made of 1Fe0.2C material, the thickness of the recast layer was a maximum of 30 µm and the samples were covered with about 70%.

### 3.5. The Analysis of TEMLamella

**Figure 9:**
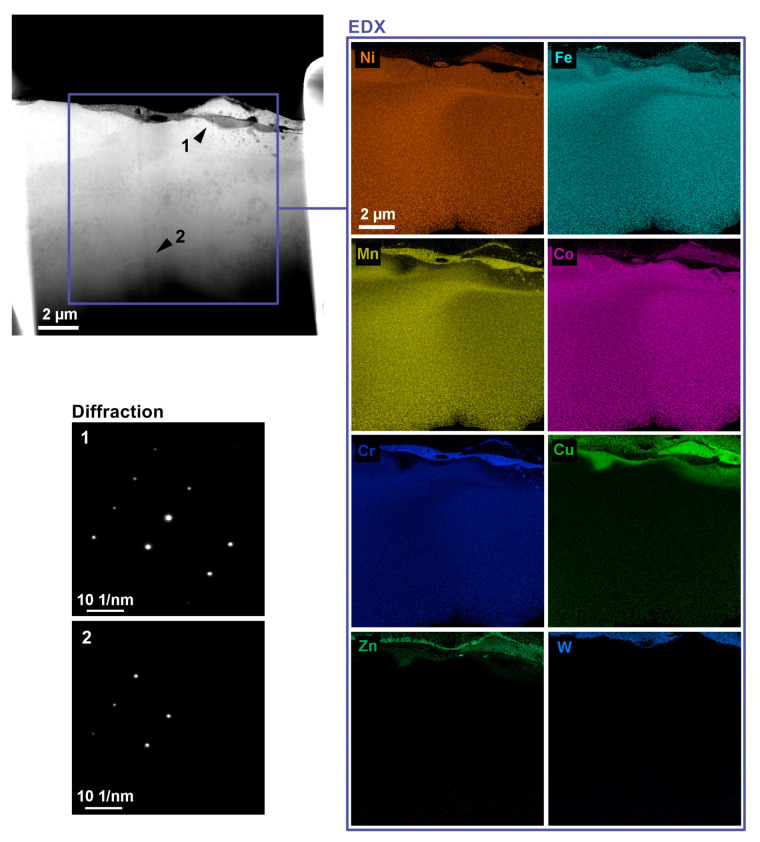
Transmission electron microscope (TEM) lamella made of 1Fe alloy, including maps of distribution of individual elements in various details and diffraction patterns.

The analysis of the manufactured TEM lamellas from Samples 20 (the samples with the lowest topography parameters produced with the setting of machine parameters: *U* = 70 V, *T_on_* = 6 µs, *T_off_* = 50 µs, *v* = 14 m/min, and *I* = 25 A) included the analysis of the chemical composition and determination of the impact of WEDM on the crystal structure of the material. EDX measurements were performed in a Titan G2 transmission electron microscope with an accelerating voltage of 300 kV in the transmission electron microscope scanning mode (STEM). The beam current was set to 0.7 nA to ensure a sufficient X-ray signal. From Figure 9, in which the TEM lamella made of 1Fe is shown, it is clear that the distribution of chemical elements was most affected to a depth of 4 µm. The upper part made of tungsten served to protect the studied layer during the formation of the lamella. In the first 2 µm, there was a reduction in the amount of manganese and chromium up to isolated edges and a strong migration of material from the wire electrode in the form of copper and zinc. At a distance of 1 µm from the workpiece surface, there was a decrease in the concentration of cobalt and nickel in the dark snake-shaped area and an increase in the concentrations of manganese, iron, and chromium. This increased concentration was manifested by an adequate decrease in the lower layer to a distance of 1 µm. In addition, in this layer, in the right part of the lamella, there was a group of circular shapes formed by the same elements as the dark area. In the rest of the lamella, the individual elements were evenly distributed. To determine the impact of WEDM on the crystal lattice of the material, it was first necessary to set the microscope to the projection mode, in which the beam size is equal to the size of the observation field, and then switch to diffraction imaging. To increase the intensity of the diffraction image, the beam current was set to 10 nA. Two photographs were taken in the diffraction image. The first was taken at a distance of 8 µm from the surface and the second at only 2 µm, which is indicated by the arrows in Figure 9. From the diffraction in Figure 2, it is clear that there was no big change due to machining; there was only a change in interplanar distances of 1%. In two of the three vectors, there was an expansion due to machining and, in the remaining one, there was a reduction, which indicates the formation of residual stress.

**Figure 10:**
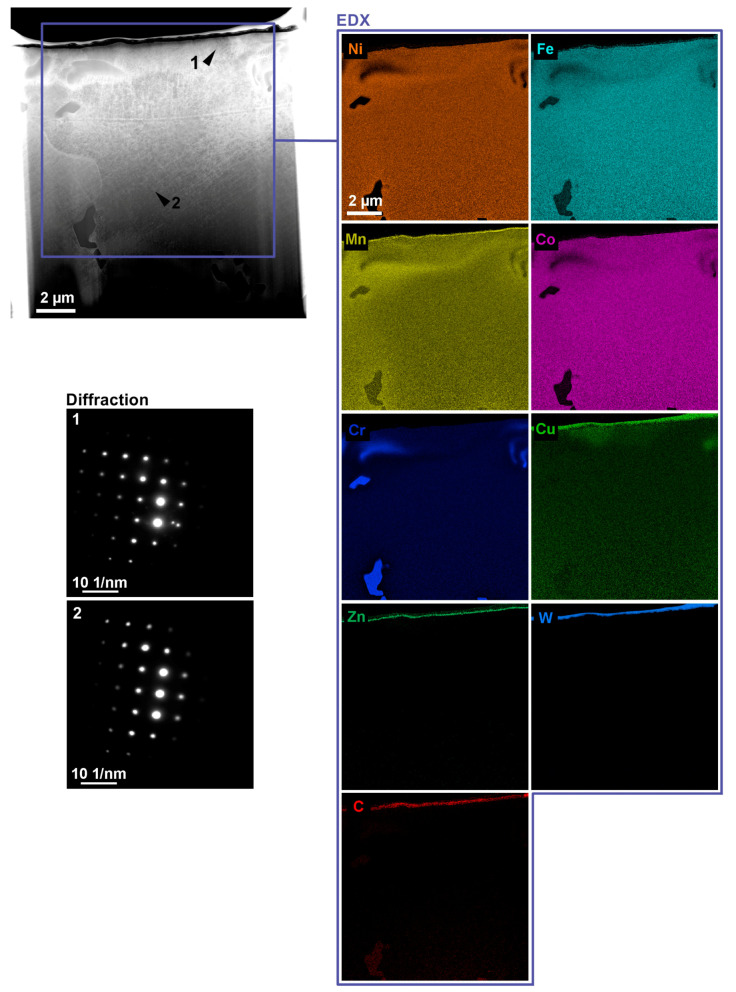
TEM lamella made of 1Fe0.2C alloy, including maps of distribution of individual elements in various details and diffraction patterns.

The study of the 1Fe0.2C lamella included measurements of the local concentration of elements (EDX) and measurements of the change in the plane in distance in diffraction imaging. EDX measurements were performed in the scanning mode of a microscope with a beam current of about 1 nA over the entire surface of the lamella. From Figure 10, it is clear that the amounts of cobalt, chromium, nickel, and iron have been reduced in the area of the recast layer. In manganese, on the other hand, there was an increase in the local concentration; in addition, migration of material from the wire electrode (copper and zinc) was detected in this area. The detected copper in the rest of the lamella was probably transferred to the sample during the preparation of the lamella from the copper holder. In the rest of the lamella, the concentration of individual elements was relatively constant except for the discovered grains composed mainly of chromium. The study of the diffraction changes of the crystal structure due to WEDM was performed in the projection mode with a current of about 8 nA with the help of a selection aperture. The diffraction pattern was obtained from the area of 8 µm and 1.5 m from the workpiece surface, while their comparison shows that there was a slight decrease in the distance of individual points, which manifested itself in increasing the distance by up to 3%. In addition, recrystallization of a new plane (point outside the regular order) was also observed on the diffraction pattern marked 1.

## 4. Conclusions

In order to study the machinability of highly entropic FeCoCrMnNi and FeCoCrMnNiC_0.2_ alloys in detail, an extensive design of experiments called “central composite design” including 66 rounds was performed, in which the influence of machine setting parameters pulse off time, gap voltage, discharge current, pulse on time, and wire speed on the cutting speed and the resulting quality of the surface and subsurface layer of the machined samples was monitored. Based on the large number of analyses performed, the following conclusions were reached:

- Sample 31 made of the material FeCoCrMnNiC0 was cut with the highest speed of 3.15 mm/min 2 with the setting of the machine parameters: *U* = 50 V, *T_on_* = 10 µs, *T_off_* = 30 µs, *v* = 14 m/min, and *I* = 35 A;
- Based on the measured outputs, a regression model was constructed describing the cutting speed of individual samples, with the model describing 97.7% of the variability of the monitored cutting speeds;
- The machine setting parameters pulse on time and discharge current have a positive effect on the cutting speed and pulse off time has a negative effect, while the alloy type itself (FeCoCrMnNi or FeCoCrMnNiC0.2) is not significant in comparison with these factors, although its effect is statistically significant;
- The lowest parameters of the surface topography (Ra 2.08 µm) were achieved for a sample made of FeCoCrMnNiC_0.2_ alloy according to the settings for Sample 20 with the setting of machine parameters: *U* = 70 V, *T_on_* = 6 µs, *T_off_* = 50 µs, *v* = 14 m/min, and *I* = 25 A;
- Regression models for the topography parameters Ra and Rz for both alloys were compiled, while the input parameters of the machine settings pulse on time and discharge current have the greatest influence on them;
- The analysis of morphology revealed the occurrence of small cracks up to 10 µm in length on the surface of all samples from both alloys, but these cracks are only occasional and it is clear from the crosssection of the sample that they have only a surface character and do not extend to the depth;
- For FeCoCrMnNi alloy samples, approximately 30% of the surface was formed by smooth craters, where there was no significant diffusion of wire electrode elements and their content is up to 3.3 wt.% of copper and 2.1 wt.% of zinc, while on the contrary, FeCoCrMnNiC_0.2_ samples were covered by a significantly larger number of smooth places, which here accounted for about 60% of the entire surface;
- Crosssections of all samples clearly excluded the presence of any subsurface defects;
- Manufactured TEM lamellas showed diffusion of elements from the wire electrode and discovered the presence of places with the increased concentration of some elements.

Given the above-mentioned conclusions obtained from the analyses, it can be clearly stated that the optimal setting of machine parameters was discovered, which allows machining of highly entropic alloys FeCoCrMnNi and FeCoCrMnNiC_0.2_ with the required surface quality without defects.

## Figures and Tables

**Table 1:** Alloy composition (mass %) and aberrations in FeCoCrMnNi high entropy alloys (HEAs) and FeCoCrMnNiC_x_ HE cast iron alloys [23].

Alloys	Alloys (abbr.)	Mass Percent (mass%)					
		Fe	Co	Cr	Mn	Ni	C
FeCoCrMnNi	1Fe	19.92	21.02	18.54	19.59	20.93	
FeCoCrMnNiC_0.2_	1Fe0.2C	19.75	20.84	18.39	19.43	20.75	0.85

**Table 2:** Limit values of machine setting parameters.

Parameter	Gap Voltage	Pulse on Time	Pulse off Time	Wire Speed	Discharge Current
	(V)	(µs)	(µs)	(m/min)	(A)
Level 1	50	6	50	10	25
Level 2	60	8	40	12	30
Level 3	70	10	30	14	35

**Table 3:** Machining parameters used in the experiment and cutting speed.

Number of Sample	Gap Voltage (V)	Pulse on Time (µs)	Pulse off Time (µs)	Wire Speed (m/min)	Discharge Current (A)	Number of Sample	Gap Voltage (V)	Pulse on Time (µs)	Pulse off Time (µs)	Wire Speed (m/min)	Discharge Current (A)
**1**	70	8	40	12	30	**18**	60	8	40	12	30
**2**	60	8	30	12	30	**19**	60	8	40	12	30
**3**	60	8	40	12	25	**20**	70	6	50	14	25
**4**	60	10	40	12	30	**21**	50	6	30	14	25
**5**	50	8	40	12	30	**22**	60	8	40	12	30
**6**	60	8	50	12	30	**23**	70	10	30	14	25
**7**	60	6	40	12	30	**24**	50	6	50	10	25
**8**	60	8	40	12	35	**25**	60	8	40	12	30
**9**	60	8	40	10	30	**26**	50	10	50	14	25
**10**	60	8	40	14	30	**27**	50	10	30	10	25
**11**	60	8	40	12	30	**28**	50	6	50	14	35
**12**	50	6	30	10	35	**29**	50	10	50	10	35
**13**	70	10	50	10	25	**30**	70	6	30	14	35
**14**	70	10	30	10	35	**31**	50	10	30	14	35
**15**	60	8	40	12	30	**32**	60	8	40	12	30
**16**	70	6	50	10	35	**33**	70	6	30	10	25
**17**	70	10	50	14	35						

**Table 4:** Contributions of individual predictors.

Source	Contribution	*p*-Value
Model	97.70%	0.000
Pulse on time (µs)	17.90%	0.000
Pulse off time (µs)	34.82%	0.000
Discharge current (A)	43.36%	0.000
Alloy	0.71%	0.000
Discharge current (A)*Discharge current (A)	0.41%	0.002
Pulse on time (µs)*Discharge current (A)	0.50%	0.001

**Table 5:** Contributions of the variability of individual predictors to the overall variability of the modelled characteristic.

Source	1Fe-Ra (µm)	1Fe-Rz (µm)	1Fe0.2C-Ra (µm)	1Fe0.2C-Rz (µm)
Model (R2)	78.61%	82.15%	94.33%	80.26%
Linear	71.77%	69.00%	88.71%	72.68%
Gap voltage (V)	7.64%	11.87%	3.47%	0.41%
Pulse on time (µs)	25.10%	35.44%	17.38%	26.73%
Pulse off time (µs)	Non-significant	1.22%	9.80%	9.56%
Discharge current (A)	39.03%	20.41%	57.81%	35.98%
Two-way interactions	6.85%	13.15%	5.62%	7.58%

**Table 6:** Analysis of chemical composition in individual places according to Figure 6 in wt. %.

Element	Place of Measurement				
	1	2	3	4	5
**Cr**	17.4	14.2	15.7	15.3	18.9
**Mn**	19.4	10.8	15.9	11.1	20.5
**Fe**	17.2	12.1	16.5	9.8	18.4
**Co**	17.5	9.1	16.7	7.5	16.3
**Ni**	16.8	8.8	15.9	6.9	18.9
**C**	3.5	7.2	6.1	13.4	3.2
**O**	5.2	14.1	7.8	22.5	3.8
**Cu**	3	20.3	3.3	9.2	
**Zn**		3.4	2.1	4.3	

**Table 7:** Analysis of chemical composition in individual places according to Figure 7 in wt. %.

Element	Place of Measurement				
	1	2	3	4	5
**Cr**	17	16.4	11.4	10.8	16.8
**Mn**	16.4	16.9	9.8	7.9	17.4
**Fe**	19	18.3	8.3	7.4	17.8
**Co**	17.6	17.5	7.7	8.3	18.3
**Ni**	16.2	15.9	9.3	10.2	16.7
**C**	3.5	3.1	15.2	16.8	2.1
**O**	4.4	6.8	18.9	14.3	7.8
**Cu**	3.6	3.1	13.7	19.5	2.7
**Zn**	2.3	1.8	5.7	4.8	0.4

## References

[1] Jameson E.C. (2001). Electrical Discharge Machining.

[2] Sidpara A.M., Malayath G. (2019). Micro Electro Discharge Machining: Principles and Applications.

[3] Gore A.S., Patil N. (2018). Wire electro discharge machining of metal matrix composites: A review. Procedia Manuf..

[4] Vates U.K. (2018). Wire-EDM Process Parameters and Optimization.

[5] Trčka T., Polzer A., Sedlák J. (2018). End mills with PCD inserts sharpened by different electrical technologies. MM Sci. J..

[6] Murty B.S., Yeh J.-W., Ranganathan S., Bhattacharjee P.P. (2019). High-Entropy Alloys.

[7] Mouralova K., Benes L., Zahradnicek R., Bednar J., Hrabec P., Prokes T., Matousek R., Fiala Z. (2018). Quality of surface and subsurface layers after WEDM aluminum alloy 7475-T7351 including analysis of TEM lamella. Int. J. Adv. Manuf. Technol..

[8] Mouralova K., Benes L., Prokes T., Bednar J., Zahradnicek R., Jankovych R., Fries J., Vontor J. (2020). Analysis of the Machinability of Copper Alloy Ampcoloy by WEDM. Materials.

[9] Mouralova K., Prokes T., Benes L., Sliwkova P. (2019). Analysis of subsurface defects occurrence in abrasion resistant Creusabro steel after WEDM including the study of morphology and surface topography. Mach. Sci. Technol..

[10] Mouralova K., Prokes T., Benes L., Bednar J. (2019). The Influence of WEDM Parameters Setup on the Occurrence of Defects When Machining Hardox 400 Steel. Materials.

[11] Mouralova K., Benes L., Bednar J., Zahradnicek R., Prokes T., Matousek R., Hrabec P., Fiserova Z., Otoupalik J. (2019). Using a DoE for a comprehensive analysis of the surface quality and cutting speed in WED-machined hadfield steel. J. Mech. Sci. Technol..

[12] Koch L., Granberg F., Brink T., Utt D., Albe K., Djurabekova F., Nordlund K. (2017). Local segregation versus irradiation effects in high-entropy alloys: Steady-state conditions in a driven system. J. Appl. Phys..

[13] Guo J., Goh M., Zhu Z., Lee X., Nai M.L.S., Wei J. (2018). On the machining of selective laser melting CoCrFeMnNi high-entropy alloy. Mater. Des..

[14] Shen W.-J., Tsai M.-H., Yeh J.-W. (2015). Machining Performance of Sputter-Deposited (Al_0.34_Cr_0.22_Nb_0.11_Si_0.11_Ti_0.22_)_50_N_50_ High-Entropy Nitride Coatings. Coatings.

[15] He J., Wang H., Huang H.L., Xu X., Chen M., Wu Y., Liu X., Nieh T., An K., Lu Z. (2016). A precipitation-hardened high-entropy alloy with outstanding tensile properties. Acta Mater..

[16] Pickering E., Muñoz-Moreno R., Stone H., Jones N. (2016). Precipitation in the equiatomic high-entropy alloy CrMnFeCoNi. Scr. Mater..

[17] Zhang F., Wu Y., Lou H., Zeng Z., Prakapenka V.B., Greenberg E., Ren Y., Yan J., Okasinski J.S., Liu X. (2017). Polymorphism in a high-entropy alloy. Nat. Commun..

[18] Laplanche G., Kostka A., Horst O., Eggeler G., George E. (2016). Microstructure evolution and critical stress for twinning in the CrMnFeCoNi high-entropy alloy. Acta Mater..

[19] Yao Y., Huang Z., Xie P., Lacey S.D., Jacob R.J., Xie H., Chen F., Nie A., Pu T., Rehwoldt M. (2018). Carbothermal shock synthesis of high-entropy-alloy nanoparticles. Science.

[20] Otto F., Dlouhý A., Pradeep K., Kuběnová M., Raabe D., Eggeler G., George E. (2016). Decomposition of the single-phase high-entropy alloy CrMnFeCoNi after prolonged anneals at intermediate temperatures. Acta Mater..

[21] Tracy C.L., Park S., Rittman D.R., Zinkle S.J., Bei H., Lang M., Ewing R.C., Mao W.L. (2017). High pressure synthesis of a hexagonal close-packed phase of the high-entropy alloy CrMnFeCoNi. Nat. Commun..

[22] Wani I.S., Bhattacharjee T., Sheikh S., Lu Y.P., Chatterjee S., Bhattacharjee P.P., Guo S., Tsuji N. (2016). Ultrafine-Grained AlCoCrFeNi2.1Eutectic High-Entropy Alloy. Mater. Res. Lett..

[23] Nagase T., Kakeshita T., Matsumura K., Nakazawa K., Furuya S., Ozoe N., Yoshino K. (2019). Development of Fe-Co-Cr-Mn-Ni-C high entropy cast iron (HE cast iron) available for casting in air atmosphere. Mater. Des..

[24] Mouralova K. (2015). Moderní Technologie Drátového Elektroerozivního Řezání Kovových Slitin. Ph.D. Thesis.

[25] Montgomery D.C. (2017). Design and Analysis of Experiments.

[26] Manoj I.V., Joy R., Narendranath S. (2020). Investigation on the Effect of Variation in Cutting Speeds and Angle of Cut during Slant Type Taper Cutting in WEDM of Hastelloy X. Arab. J. Sci. Eng..

[27] ISO (1997). Geometrical Product Specifications (GPS)—Surface Texture: Profile Method—Terms, Definitions and Surface Texture Parameters.

[28] Tosun N., Çogun C., Inan A. (2003). The Effect of Cutting Parameters on Workpiece Surface Roughness in Wire EDM. Mach. Sci. Technol..

[29] Aspinwall D., Soo S., Berrisford A., Walder G. (2008). Workpiece surface roughness and integrity after WEDM of Ti–6Al–4V and Inconel 718 using minimum damage generator technology. CIRP Ann..

[30] McGeough J.A. (1988). Advanced Methods of Machining.

[31] Newton T.R., Melkote S.N., Watkins T., Trejo R.M., Reister L. (2009). Investigation of the effect of process parameters on the formation and characteristics of recast layer in wire-EDM of Inconel 718. Mater. Sci. Eng. A.

[32] Kumar A., Kumar V., Kumar J. (2016). Surface crack density and recast layer thickness analysis in WEDM process through response surface methodology. Mach. Sci. Technol..

[33] Rupajati P., Soepangkat B.O.P., Pramujati B., Agustin H.K. (2014). Optimization of Recast Layer Thickness and Surface Roughness in the Wire EDM Process of AISI H13 Tool Steel Using Taguchi and Fuzzy Logic. Appl. Mech. Mater..

[34] Azam M., Jahanzaib M., Abbasi J.A., Abbas M., Wasim A., Hussain S. (2016). Parametric analysis of recast layer formation in wire-cut EDM of HSLA steel. Int. J. Adv. Manuf. Technol..

[35] Huang C., Hsu F., Yao S. (2004). Microstructure analysis of the martensitic stainless steel surface fine-cut by the wire electrode discharge machining (WEDM). Mater. Sci. Eng. A.

[36] Klocke F., Hensgen L., Klink A., Ehle L., Schwedt A. (2016). Structure and Composition of the White Layer in the Wire-EDM Process. Procedia CIRP.

[37] Kumar A., Kumar V., Kumar J. (2013). Investigation of microstructure and element migration for rough cut surface of pure titanium after WEDM. Int. J. Microstruct. Mater. Prop..

